# Calculation and Identification of the Aerodynamic Parameters for Small-Scaled Fixed-Wing UAVs

**DOI:** 10.3390/s18010206

**Published:** 2018-01-13

**Authors:** Jieliang Shen, Yan Su, Qing Liang, Xinhua Zhu

**Affiliations:** 1 School of Mechanical Engineering, Nanjing University of Science and Technology, Nanjing 210094, China; perfect.sjlchg2008@163.com; 2 School of Computer Technologies and Control, ITMO University, St. Petersburg 197101, Russia; liangqing1688@gmail.com

**Keywords:** aerodynamic parameters, semi-empirical aerodynamic coefficient modeling, parameters identification, EKF, real flight tests

## Abstract

The establishment of the Aircraft Dynamic Model (ADM) constitutes the prerequisite for the design of the navigation and control system, but the aerodynamic parameters in the model could not be readily obtained especially for small-scaled fixed-wing UAVs. In this paper, the procedure of computing the aerodynamic parameters is developed. All the longitudinal and lateral aerodynamic derivatives are firstly calculated through semi-empirical method based on the aerodynamics, rather than the wind tunnel tests or fluid dynamics software analysis. Secondly, the residuals of each derivative are proposed to be identified or estimated further via Extended Kalman Filter (EKF), with the observations of the attitude and velocity from the airborne integrated navigation system. Meanwhile, the observability of the targeted parameters is analyzed and strengthened through multiple maneuvers. Based on a small-scaled fixed-wing aircraft driven by propeller, the airborne sensors are chosen and the model of the actuators are constructed. Then, real flight tests are implemented to verify the calculation and identification process. Test results tell the rationality of the semi-empirical method and show the improvement of accuracy of ADM after the compensation of the parameters.

## 1. Introduction

In contrast to the large-scaled high-altitude long-term Unmanned Aerial Vehicles (UAVs) for remote surveillance and combat, the small-scaled fixed-wing UAVs, like “Raven” RQ-11 and Sand Hawk of the US, or “Rainbow-802” of China, have unique superiority in civilian and military fields. With the light weight, small volume and simple take-off, small UAVs could execute tasks like the close-range monitoring in a covert and flexible way [1]. The establishment of Aircraft Dynamic Model (ADM) should be accomplished first before any task designing. It contains the computation of aircrafts’ structural parameters, position of the center of gravity, moment of inertia, aerodynamic parameters and the modeling of actuators.

For small-scaled fixed-wing UAVs, the structural parameters, like the wing span *l*, mean aerodynamic chord $\bar{c}$ and the reference area *S*, could be measured precisely. The thrust, driven by the propeller, can be determined by the diameter of the propeller *D*, rotation rate $n_{P}$ and airspeed $V_{a}$ [2]. However, the aerodynamic parameters, covering stability derivatives and the control derivatives, are relatively hard to get, which is crucial in calculating the force and moment. Stability derivatives involve partial derivatives with respect to states and control derivatives involve partial derivatives with respect to control inputs. The stability derivatives could be further divided into static stability derivatives for derivatives associated with air-relative velocity quantities and the dynamic stability derivatives for derivatives associated with angular rates and unsteady aerodynamics [3]. Generally, the aerodynamic parameters are analyzed through wind tunnel tests or CFD software, which are not appropriate for small ones. Wind tunnel test is high-cost and time-consuming, most of all, the results are not accurate in the flight condition of low Reynolds number. And CFD software analysis usually has heavy work on the 3D model meshing, and the gap effect as well as the frictional drag is often neglected [4,5]. DATCOM, introduced by the US Air Force, is another choice for the parameter calculation. On the basis of the huge flight database, all the aerodynamic coefficients could be obtained when the required parameters are imported. But DATCOM does have drawbacks. The input parameters are quite complex and the analysis will somehow be affected by the computational interval of the angle-of-attack α, resulting in limited accuracy for small UAVs [6,7].

Considering the special aerodynamic configuration and flight condition of the small UAVs, the semi-empirical method based on the aerodynamic theory is proposed here, by means of which the longitudinal and lateral derivatives could be calculated step by step according to the semi-empirical formulas or diagrams. Semi-empirical method was also mentioned in Arifianto’s research [8]. The reason why it is called ”Semi-empirical” is that the formulas and diagrams used are actually the combination of the fundamental aerodynamics and empirical summary from abundant flight tests. For instance, the diagram for calculate the lift curve slope of wing $C_{W}^{\alpha }$ is fitted with the flight data according to the theoretical relationship with the geometrical shape of the wing and the Mach number. Monographs [9,10,11,12,13,14] introduce the basic aerodynamic theory of different aircrafts flying under different circumstances, and describe the detailed procedure of calculating all the aerodynamic derivatives. Specifically, the aerodynamic analysis of small-scaled aircrafts is often simplified for quite small Mach number, plain aerodynamic structure and small angle-of-attack assumption. So, this kind semi-empirical method could rationally calculate the initial parameters in a small amount of computation with low cost and it will be adopted in the paper. Although the semi-empirical analysis is an effectively feasible method, the accuracy of the parameters is sometimes suspicious due to the structure simplification and the changing of the flight condition. After the theoretical calculation, the aerodynamic parameters are nearly all constants, which is understandable in the small Mach number condition and small angle-of-attack assumption. It means that the method owns poor real-time performance. Therefore, the identification process is carried out to further compensate the error of aerodynamic parameters with the real-time flight data.

Monographs [3,15] synthesize the basic issues of system identification for fixed-wing aircrafts or rotorcrafts, including aircraft’s mathematical model, estimation theories, classification of identification methods as well as engineering practices, like experiment design, data compatibility check and data analysis. Generally, there exists many approaches to identify or estimate the error of the aerodynamic parameters, and it could be classified into off-line and on-line way. Off-line way consists of the formula error method and the output error method, the former is based on the Least Square (LS) while the latter based on the Maximum Likelihood Method(MLE). And off-line way could be operated either in the the time domain or the frequency domain [16,17]. Neural network is also chosen as the structure to describe or identify the dynamic characteristics of aircraft [18,19]. All the methods above own a large amount of computation and call for the whole accurate measurements corresponding to the system states. Morelli [20] focuses on the aerodynamic model identification of the combat aircraft like F-16 using MLE in the frequency domain, and the measurements used are the 6 outputs of the IMU and the aerodynamic states like *V${}_{a}$*, α and β, which requires high-precision IMUs and expensive sensors for α, β detection. But it is quite an expectation for small-loaded aircrafts. Dorobantu’s research [21,22] exhibit the system identification for small, low-cost, fixed-wing UAVs, which usually carry lightweight devices, like the MEMS IMUs, but the accuracy of the measurement is usually hard to guarantee.

On-line identification, or the real-time estimation, could handle the problems above. Based on the linear or non-linear Kalman Filter, errors of the aerodynamic parameters are expanded into the motion states, and then estimated to some extent in a real time. Garcia [23] introduces that EKF method breaks the limitation of the linearity of the system and the estimation results could be variant. But, the inaccuracy of the statistical characteristics of noise and the observability issue affect the estimating results. According to [24], maneuvering motion, like the changing of the acceleration and angular velocity, will be able to increase the observability of system states. Hence, the specific maneuvers excited by the changing of the inputs of the actuators definitely improve the observability of each expanded states [25]. After the categorization of current identification methods for different kinds of aircrafts equipped with different sensors, Hoffer [26] proposed that the Square-root UKF would be a good choice for the on-line identification. Girish [27] employs both EKF and UKF to estimate the aerodynamic parameters with the flight data, and it is pointed out that although UKF owns a theoretically better precision, EKF shows the same performance with less computation. Therefore, EKF is utilized in the paper to estimate and compensate the error of all the longitudinal and lateral aerodynamic derivatives. And the observations are the relatively accurate airborne integrated INS/GPS attitude and velocity. Meanwhile, maneuvering flight path is designed for the promotion of observability. The identified parameters could be used to calculate the navigation results with the dynamic and kinematic model of aircraft, or the motion model, then aiding the low-grade INS [28]. So the precision of identification results could be evaluated by comparing the navigation results with that of INS/GPS. It is quite different for the evaluation of the identification of second-order transfer function.

The outline of this paper is as follows. In Section 2, the aerodynamic characteristics of the small-scaled fixed-wing UAVs are summarized and all the parameters are calculated semi-empirically. In Section 3, we present the process of the identification of the aerodynamic parameters in details, and also the observability analysis is discussed. Section 4 selects the airborne sensors and the data acquisition board, then describes the modeling of the propeller and the control surfaces as a test preparation. In Section 5, the results of the flight tests are demonstrated as a verification. At last, the paper is concluded in Section 6.

## 2. Semi-Empirical Calculation

**Figure 1:**
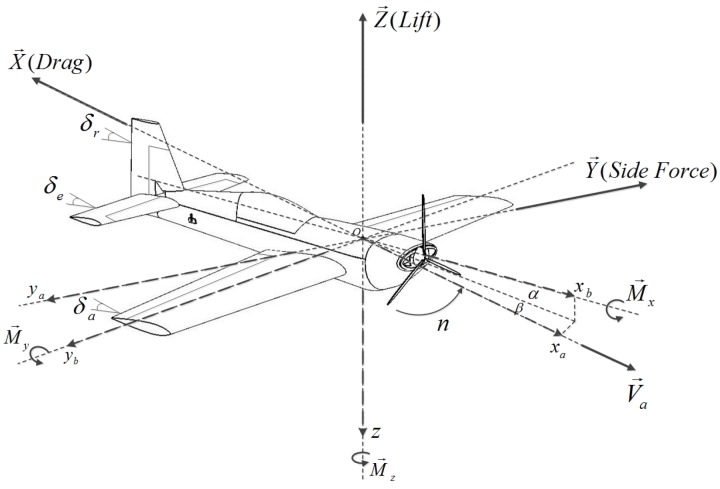
Aerodynamic Forces & Moments.

Aerodynamic parameters reside in the aerodynamic forces and moments acting on the aircraft, containing the lift *Z*, drag *X* and pitch moment $M_{y}$ in the longitudinal channel and the side force *Y*, roll moment $M_{x}$ and yaw moment $M_{z}$ in the lateral channel, shown in Figure 1. It is a typical fixed-wing aircraft with a conventional configuration. Table 1 lists the formulas for the calculation of the thrust driven by the propeller and all the aerodynamic forces and moments, which are the product of the dynamic pressure *Q* ($Q=\rho V_{a}^{2}/2$), reference area *S*, reference size *l* or $\bar{c}$ and the non-dimensional coefficients $C_{i}$ or $m_{i}$, i=X,Y,Z. As seen in the formulas, $C_{i}$ or $m_{i}$ consists of several non-dimensional and dimensional parameters, namely the aerodynamic derivatives, which are exactly the targeted parameters in this paper.

Aerodynamic derivatives are determined by the flight condition, aerodynamic layout and structural characteristic. Reynolds number of small UAVs, around 200,000, belongs to low Reynolds number. Laminar separation occurs and the aerodynamic efficiency decreases. For instance, the relationship between the lift coefficient and α, namely $C_{Z}^{\alpha }$, would be nonlinear as the α increases. Hysteresis effect would happen around the stall angle resulting in the abnormal change of $C_{Z}^{\alpha }$ [29]. Given the condition of small Mach number Ma, the real-time changing of the aerodynamic derivatives caused by the velocity could be neglected, and the assumption of small α or β could make the derivatives, for example $C_{Z}^{\alpha }$, $C_{Y}^{\beta }$, constants [12,13]. Therefore, the nonlinear aerodynamic derivatives, like $C_{Z}^{\alpha^{2}}$, $C_{Z}^{\alpha q}$, $C_{Z0}\;\left(\alpha ,\beta \right)$, are neglected for the ignoring of large-amplitude maneuvers and high angle-of-attack, which could be a huge simplification. And dynamic derivatives referenced to the $\dot{\alpha }$, $\dot{\beta }$ could be small and also neglected, just as shown in Table 1. Moreover, small-scaled UAVs usually own a conventional configuration, namely wing-body-horizontal tail-vertical tail structure, and the sweepback angle χ or dihedral angle $\psi_{d}$ doesn’t exist in the wing designing. So the complexity of the aerodynamic analysis is reduced especially for the lateral channel. And the wing of small UAV functions predominately in the aerodynamic performance, so substituting the wing for the whole aircraft could be acceptable for the lift or pitch moment parameter calculation. The detailed process of calculating all the aerodynamic derivatives will be analyzed then.

### 2.1. Longitudinal Channel Analysis

Total lift of an aircraft is the collective effect of the wing, body and horizontal tail. With regard to the small-scaled fixed-wing aircraft, wing constitutes the main source of the lift. And there often exists interaction between the wing and body. As depicted in Table 1, lift coefficient consists of $C_{Z0}$, $C_{Z}^{\alpha }$ and $C_{Z}^{\delta_{e}}$. Zero lift coefficient, $C_{Z0}$, is determined by the airfoil camber, and the incidence angle of the wing $\varphi_{W}$ and horizontal tail $\varphi_{S}$, which means that lift still exists when the angle-of-attack is zero. $C_{Z}^{\alpha }$, known as the slope of lift curve, fuses the separate cantilever-wing slope $C_{ZCWs}^{\alpha }$, the separate body slope $C_{ZBs}^{\alpha }$, the separate horizontal tail slope $C_{ZHTs}^{\alpha }$ and all the mutual effect between each of them. In details, $C_{ZCWs}^{\alpha }$, $C_{ZHTs}^{\alpha }$ could be computed with the same diagram in [13,14], both of which are the function of the structural parameters of the wing and the Mach number Ma. While the lift slope of body could be divided into the conical or ogive head, the cylindrical body and the shrinking tail part. $C_{Z}^{\delta_{e}}$ concerns with the aerodynamic efficiency $\eta_{e}$ and the size of the elevator.

Lift of the wing-body part $Z_{WB}$ is the sum of the lift of separate body $Z_{Bs}$ and the lift caused by the existence of the cantilever-wing $Z_{CW}$, while the latter contains the lift of separate cantilever-wing $Z_{CWs}$, the interference lift from the body to the cantilever-wing $Z_{B-CW}$ and the opposite lift $Z_{CW-B}$. Interference factors [13,14], $K_{\alpha \alpha }$, $K_{\varphi 0}$, are introduced to describe the mutual effect numerically, both of which concerns with the shape of the wing and the diameter-to-span ratio $\bar{D}$. Generally, when we have the condition that the aspect ratio λ is large enough (λ⩾5) and $\bar{D}$ is small enough ($\bar{D}⩽0.1$), neglecting the mutual effect hardly matters. In the linear range, the lift slope of the wing-body part are shown in (1) and (2).

(1) $$C_{ZWB}^{\alpha }=C_{ZBs}^{\alpha }\frac{S_{B}}{S}+C_{ZCWs}^{\alpha }K_{\alpha \alpha }\frac{S_{CW}}{S}$$

(2) $$C_{ZWB}^{\varphi }=C_{ZCWs}^{\alpha }K_{\varphi 0}\frac{S_{CW}}{S}$$

The airflow block factor $k_{HT}$ and the down wash angle ε, caused by the wing part, should be taken into account when analyzing the lift of the horizontal tail. The block factor $k_{HT}$ ($k_{HT}=0.85$) should be multiplied to the dynamic pressure while the down wash angle ε should be eliminated to obtain the real angle-of-attack of the horizontal tail $\alpha_{HT}$. The down wash angle after the wing-body part $\varepsilon_{WB}$ are nearly linear to the α and the incidence angle of the wing, so the related derivatives are calculated in (3) and (4), in which $\varepsilon_{W}^{\alpha }$, the down wash caused by the separate wing could be calculated in [13,14]. Thus, the lift coefficients could be deduced in Formulas (5)–(7), among which ${\left(K_{\alpha \alpha }\right)}_{HT}$ and ${\left(K_{\varphi 0}\right)}_{HT}$ are the interference factor between the body and the horizontal tail.

(3) $$\varepsilon_{WB}^{\alpha }=K_{\alpha \alpha }\frac{S_{CW}}{S}\frac{C_{ZCWs}^{\alpha }}{C_{ZWs}^{\alpha }}\varepsilon_{W}^{\alpha }$$

(4) $$\varepsilon_{WB}^{\varphi }=K_{\varphi 0}\frac{S_{CW}}{S}\frac{C_{ZCWs}^{\alpha }}{C_{ZWs}^{\alpha }}\frac{\varepsilon_{W}^{\alpha }}{1-\bar{D}}$$

(5) $$C_{Z0}=C_{ZWB}^{\varphi }\varphi_{W}+C_{ZHTs}^{\alpha }{\left(K_{\varphi 0}\right)}_{HT}\left(\varphi_{S}-\varepsilon_{WB}^{\varphi }\varphi_{W}\right)k_{HT}\frac{S_{HT}}{S}$$

(6) $$C_{Z}^{\alpha }=C_{ZWB}^{\alpha }+C_{ZHTs}^{\alpha }{\left(K_{\alpha \alpha }\right)}_{HT}\left(1-\varepsilon_{WB}^{\alpha }\right)k_{HT}\frac{S_{HT}}{S}$$

(7) $$C_{Z}^{\delta_{e}}=C_{ZHT}^{\delta_{e}}k_{HT}\frac{S_{HT}}{S}$$

Generally, aircraft drag contains the parasitic drag and the induced drag, neglecting the wave drag in supersonic flight. Parasitic drag usually consists of friction drag, form drag and interference drag, among which the friction drag are the main source of the parasitic drag in the low speed flight. Induced drag is caused by the lift of the wing. Under the small Mach number and small angle-of-attack assumption, the drag coefficient $C_{X}$ is separated into zero lift coefficient $C_{X0}$ and the lift-induced drag coefficient $C_{Xi}$, depicted as the parabolic formula in Table 1. $C_{X0}$ mainly contains the friction part and the due-to-lift part in the parasitic drag is neglected. So $C_{X0}$ is calculated with the surface friction coefficient $c_{f}$ and correction factor η [13,14]. $C_{Xi}$, consistent with the formation $AC_{Z}^{2}$, is calculated considering the wing-body part according to [13,14], in which $C_{Xi}$ of wing are closely related to the wing’s shape and $C_{Xi}$ of body is proportional to $\alpha^{2}$.

Assuming that the vector of thrust points through the center of gravity, the pitch moments are primarily caused by the lift of different parts. Prior to calculating the pitch moment coefficients, the action point of the lift, namely the aerodynamic center, should be analyzed, which covers the analysis of the wing, body, wing-body and the horizontal tail. Similar to the lift, the location of the aerodynamic center of the separate wing and horizontal tail $x_{pW}$, $x_{pHT}$ could be obtained by the same diagram in [13,14] according to the size and shape. Referenced to the vertex in the front, the location of aerodynamic center is usually positive. But for the body part, $x_{pB}$ could be negative as the tail generates large minus pitch moment. The location of the wing-body part ${\left(x_{pWB}\right)}_{\alpha \alpha }$, ${\left(x_{pWB}\right)}_{\varphi 0}$ could also be computed with the factor $K_{\alpha \alpha }$, $K_{\varphi 0}$. For the elevator, $x_{pHT\delta_{e}}$ locates at the leading edge of the control surface.

Along with the aerodynamic center, the aerodynamic focus is also introduced to reflect the longitudinal stability. The location of aerodynamic focus $x_{F}$, only determined by the angle-of-attack, are related to the longitudinal static stability derivative $m_{y}^{\alpha }$ and the location of the center of gravity $x_{G}$, depicting in (8). It is explained that when the $m_{y}^{\alpha }$ is negative ($x_{G}<x_{F}$) , the aircraft is statically stable. While the aircraft deviates from its equilibrium position, the pitching moment makes itself back to the equilibrium automatically. Three static aerodynamic derivatives $m_{Z0}$, $m_{Z}^{\alpha }$, $m_{Z}^{\delta_{e}}$ are deduced in (9)–(11). For the dynamic derivative, the damping moment coefficient $m_{y}^{{\bar{\omega }}_{y}}$ caused by the non-dimensional ${\bar{\omega }}_{y}$ should be taken into account to strengthen the stability which could be obtained in [14].

(8) $$m_{y}^{\alpha }=C_{Z}^{\alpha }\frac{x_{G}-x_{F}}{\bar{c}}$$

(9) $$m_{y0}=C_{ZWB}^{\varphi }\varphi_{W}\frac{x_{G}-{\left(x_{pWB}\right)}_{\varphi 0}}{\bar{c}}+C_{Z0HT}k_{HT}\frac{S_{HT}}{S}\frac{x_{G}-x_{pHT}}{\bar{c}}$$

(10) $$m_{y}^{\alpha }=C_{ZBs}^{\alpha }\frac{S_{B}}{S}\frac{x_{G}-x_{pB}}{\bar{c}}+C_{ZWB}^{\alpha }\frac{x_{G}-{\left(x_{pWB}\right)}_{\alpha \alpha }}{\bar{c}}+C_{ZHT}^{\alpha }k_{HT}\frac{S_{HT}}{S}\frac{x_{G}-x_{pHT}}{\bar{c}}$$

(11) $$m_{y}^{\delta_{e}}=C_{ZHT}^{\delta_{e}}k_{HT}\frac{S_{HT}}{S}\frac{x_{G}-x_{pHT\delta_{e}}}{\bar{c}}$$

### 2.2. Lateral Channel Analysis

The aerodynamic analysis in the lateral channel is comparatively more complicated concerning the coupling between the roll and yaw moments. But there are some easy ways to simplify the process. Firstly, side force *Y* mainly stems from the body and the vertical tail. When the aircraft rotates around the x-axis for 90${}^{{}^{\circ}}$, *Y* is just similar to the lift *Z*. As a result, the angle of sideslip β resembles the angle-of-attack α and the deflection angle of the rudder $\delta_{r}$ resembles that of elevator $\delta_{e}$. Then, the aerodynamic derivatives $C_{Y}^{\beta }$, $C_{Y}^{\delta_{r}}$ could be deduced. Similarly, the calculation of the yaw moment derivatives could be operated after the rotation of 90${}^{{}^{\circ}}$ and at this time, the side force *Y* is the main cause of the yaw moment. The static derivatives $m_{z}^{\beta }$, $m_{z}^{\delta_{r}}$ and the dynamic derivative $m_{z}^{{\bar{\omega }}_{z}}$ are computed, among which $m_{z}^{\beta }$ ($m_{z}^{\beta }<0$) determines the stability of yawing channel [13,14].

Among the roll moment derivatives, $m_{x}^{\beta }$ is an important derivative that concerns with the α, β, the aerodynamic configuration and structural size, such as the dihedral angle $\psi_{d}$, sweepback angle $\chi_{0.5}$, the wing-body interaction, the wing tip characteristic and so on, depicting in (12). The aileron plays a vital role in rolling the aircraft and the relevant control derivative $m_{x}^{\delta_{a}}$ could be obtained in (13), in which $\eta_{a}$ is the aerodynamic efficiency of the aileron. The damping roll derivative $m_{x}^{{\bar{\omega }}_{x}}$ could also be obtained just like the damping one in the yaw moment. There does exist coupling effects between the yaw and roll moment, described by the relevant derivatives $m_{z}^{\delta_{a}}$, $m_{x}^{\delta_{r}}$, $m_{z}^{{\bar{\omega }}_{x}}$, $m_{x}^{{\bar{\omega }}_{z}}$. They are usually small but ought to be considered according to the specific situation [13,14].

(12) $${\left(m_{x}^{\beta }\right)}_{*}={\left(m_{x}^{\beta }\right)}_{\psi_{d}}+{\left(m_{x}^{\beta }\right)}_{WB}+{\left(m_{x}^{\beta }\right)}_{VT}+\left[{\left(\frac{\partial^{2}m_{x}}{\partial \alpha \partial \beta }\right)}_{\chi_{0.5}}+{\left(\frac{\partial^{2}m_{x}}{\partial \alpha \partial \beta }\right)}_{Wtip}+{\left(\frac{\partial^{2}m_{x}}{\partial \alpha \partial \beta }\right)}_{\varepsilon }\right]\alpha +{\left(\frac{\partial^{2}m_{x}}{\partial \delta_{e}\partial \beta }\right)}_{\varepsilon }\delta_{e}$$

(13) $$m_{x}^{\delta_{a}}=C_{ZWs}^{\alpha }\eta_{a}\left(\frac{1}{C_{Z}^{\alpha }}\cdot \frac{m_{x}^{\beta }}{\psi_{d}}\right)$$

## 3. Parameter Identification

After the semi-empirical analysis, the initial aerodynamic parameters are obtained, but they are not accurate enough for further research. An identification process is necessary to correct the parameters. The nonlinear dynamic and kinematic differential equations of $V^{b}$, $\omega^{b}$, and Euler angles are to be used as the system equations in the identification process, shown in (14)–(16) , in which a non-rotating and flat earth is assumed. Considering the possible fast dynamic change of the motion states, the error states are chosen as system states, $X_{M}={\left[\delta u,\delta v,\delta w,\delta p,\delta q,\delta r,\delta \phi ,\delta \theta ,\delta \psi \right]}^{T}$. The error of aerodynamic parameters $X_{C}$ are expanded into the system state, depicted in (17). Here, $X_{C}$ are assumed as constants given the condition of small Mach number and angle-of-attack, and they are exactly the parameters to be estimated and then compensated to the initial value . The linearization of 1st-order Taylor Series approximation of the nonlinear model is operated and the related Jacobian matrix *F* is formulated in (18), in which *F* could be partitioned into $F_{M}$, $F_{C}$ and the zero matrix. Adding up the related noise $W_{9\times 1}$, the linearized system equation is shown in (19).

(14) $${\dot{V}}^{b}=C_{n}^{b}g^{n}+\left(F_{T}+C_{a}^{b}F_{A}\right)/m-\omega^{b}\times V^{b}$$

(15) $${\dot{\omega }}^{b}=I^{-1}\left(M-\omega^{b}\times I\omega^{b}\right)$$

(16) $$\left(\begin{array}{c} \dot{\phi }\\ \dot{\theta }\\ \dot{\psi } \end{array}\right)=\left[\begin{array}{ccc} 1 & \tan\theta \sin\phi & \tan\theta \cos\phi \\ 0 & \cos\phi & -\sin\phi \\ 0 & \frac{\sin\phi }{\cos\theta } & \frac{\cos\phi }{\cos\theta } \end{array}\right]\left(\begin{array}{c} p\\ q\\ r \end{array}\right)$$

(17) $$\begin{array}{c} X=[\delta u,\delta v,\delta w,\delta p,\delta q,\delta r,\delta \phi ,\delta \theta ,\delta \psi ,\\ \;\;\;\;\;\;\;\;\delta C_{Z0},\delta C_{Z}^{\alpha },\delta C_{Z}^{\delta_{e}},\delta C_{X0},\delta C_{XA},\delta m_{y0},\delta m_{y}^{\alpha },\delta m_{y}^{\delta_{e}},\delta m_{y}^{{\bar{\omega }}_{y}},\\ \;\;\;\;\;\;\;\delta C_{Y}^{\beta },\delta C_{Y}^{\delta_{r}},\delta m_{z}^{\beta },\delta m_{z}^{\delta_{r}},\delta m_{z}^{{\bar{\omega }}_{z}},\delta m_{z}^{{\bar{\omega }}_{x}},\delta m_{x}^{\beta },\delta m_{x}^{\delta_{a}},\delta m_{x}^{\delta_{r}},\delta m_{x}^{{\bar{\omega }}_{x}},\delta m_{x}^{{\bar{\omega }}_{z}}{]}_{29\times 1}^{T} \end{array}$$

(18) $$F=\left[\begin{array}{cc} {F_{M}}_{9\times 9} & {F_{C}}_{9\times 20}\\ 0_{20\times 9} & 0_{20\times 20} \end{array}\right]$$

(19) $$\dot{X}=FX+W$$

There are 20 aerodynamic parameters in the aircraft dynamic model according to Table 1. All the derivatives are directly fused with the airspeed $V_{a}$ residing in the dynamic pressure *Q*. It could be excited through acceleration or deceleration by changing the rotation rate of the propeller. Some derivatives have close relations with the aerodynamic angle α, β, like $C_{Z}^{\alpha }$, $m_{y}^{\alpha }$, $C_{Y}^{\beta }$, $m_{x}^{\beta }$, and the damping derivatives like $m_{y}^{{\bar{\omega }}_{y}}$, are concerned with the rotation rate of the aircraft. All of them are supposed to be stimulated by the uniform or non-uniform changing of the attitude. And the rest derivatives are directly related to the deflection angle of the control surfaces $\delta_{e}$, $\delta_{r}$, $\delta_{a}$.

As strong nonlinearity exists among the motion states, depicted in (14)–(16), and the number of invariant targeted derivatives are too many, it is not easy to compensate all of them correctly at the same time. Different parameters may have different impact on the model, and the observability is also different. Lift coefficient $C_{Z}$ is taken as an example to analyze the observability of each aerodynamic derivatives, depicted in (20). With the observations of non-gravitational acceleration and the thrust, the left hand Z is potentially calculated according to (14). Right hand of the equation is a sum formation, and it is easier to distinguish each element with different characteristics. So the aerodynamic derivative $C_{Z0}$, $C_{Z}^{\alpha }$ and $C_{Z}^{\delta_{e}}$ could be observed just when the angle-of-attack and deflection angle $\delta_{e}$ vary differently, namely they have distinguishing frequency spectrum. The analysis could be generalized into the moment coefficients, like the roll moment in (21). Also it is not easy to tell apart $\omega_{x}$ from $\omega_{z}$, so $m_{x}^{{\bar{\omega }}_{x}}$, $m_{x}^{{\bar{\omega }}_{z}}$ may not be separated totally, same theory for $m_{x}^{\delta_{a}}$, $m_{x}^{\delta_{r}}$. Therefore, for the similar derivatives, they could be observed when the corresponding actuators are excited at different time.

(20) $$Z=QSC_{Z}=\frac{1}{2}\rho S\left(V_{a}^{2}C_{Z0}+V_{a}^{2}C_{Z}^{\alpha }\alpha +V_{a}^{2}C_{Z}^{\delta_{e}}\delta_{e}\right)$$

(21) $$M_{x}=QSlm_{x}\;=\frac{1}{2}\rho Sl\left(V_{a}^{2}m_{x}^{\beta }\beta +V_{a}^{2}m_{x}^{\delta_{a}}\delta_{a}+V_{a}^{2}m_{x}^{\delta_{r}}\delta_{r}+\frac{l}{2}V_{a}m_{x}^{{\bar{\omega }}_{x}}\omega_{x}+\frac{l}{2}V_{a}m_{x}^{{\bar{\omega }}_{z}}\omega_{z}\right)$$

To sum up theoretically, if all the aerodynamic parameters are to be observed, the aerodynamic forces and moments are calculated firstly and the observations of acceleration and angular velocity are necessary, referring to the dynamic Equations (14) and (15). But, for small-scaled fixed-wing aircraft, it is hard for the MEMS-IMU to provide precise observations of acceleration and angular velocity on account of the unpredictable drift or bias. As MEMS-IMU integrated with GPS is the most common airborne navigation devices, the integrated navigation results could be adopted as measurements for further compensation of the aerodynamic coefficients. The acceleration and angular velocity could be deduced by the first order derivative, instead of the outputs of MEMS-IMU. So, it is reasonable to accomplish the identification process just with the observations of velocity and attitude. Now, the differences between the relatively accurate velocity and attitude from the INS/GPS fusion(subscript ”SF”) and that from the integral of the aircraft motion model of Equations (14)–(16) (subscript ”AMM”) are used as measurements, shown in (22). $u_{SF}$, $v_{SF}$, $w_{SF}$ are deduced from the INS/GPS ground velocity $V_{N}$, $V_{E}$, $V_{D}$ through a coordinate transformation by multiplying the rotation matrix $C_{n}^{b}$ computed with Euler angles. Then, the identification process will be accomplished by EKF.

(22) $$Z=\left(\begin{array}{c} \delta u\\ \delta v\\ \delta w\\ \delta \phi \\ \delta \theta \\ \delta \psi \end{array}\right)=\left(\begin{array}{c} u_{AMM}-u_{SF}\\ v_{AMM}-v_{SF}\\ w_{AMM}-w_{SF}\\ \phi_{AMM}-\phi_{SF}\\ \theta_{AMM}-\theta_{SF}\\ \psi_{AMM}-\psi_{SF} \end{array}\right)$$

The maneuvering flight is also indispensable to excite all the motion states and the inputs of the actuators to increase the observability of all the aerodynamic derivatives. Relevant researches [3,8,15] introduce the specific changes of the elevator, rudder and aileron to excite the expecting maneuvering, like dutch roll, bank-to-bank and the short period motion of the aircraft, which is an effective approach to identify the parameters in the time domain. Here in the paper, a maneuvering flight is designed, including acceleration and deceleration, rolling, the rise and fall when circling around.

## 4. Experimental Preparation

**Figure 2:**
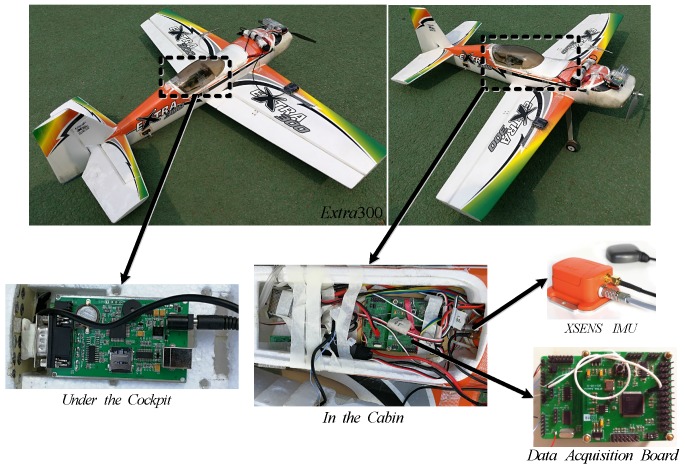
Flight Test Devices.

**Figure 3:**
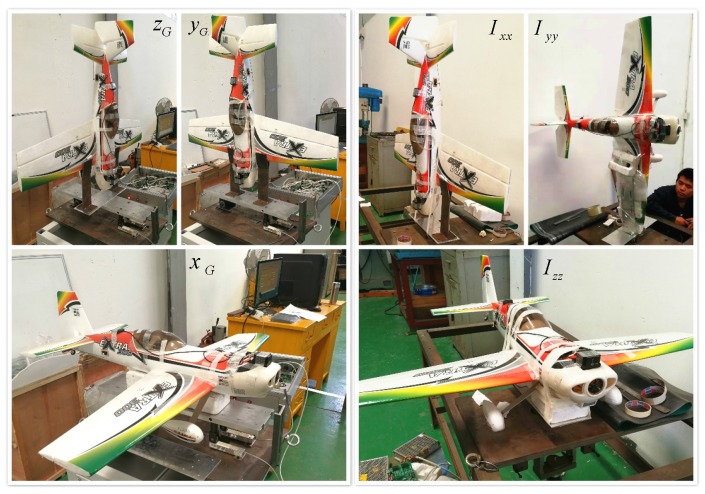
Characteristic Parameter Test.

A model UAV called Extra300, shown in Figure 2, is employed to carry out the real flight test. Two goals are about to achieve in the practical tests: (1) Rationality of the empirically calculated aerodynamic coefficients should be validated. (2) Parameters identification is operated and the improvement should be proved compared with the semi-empirical results. For the empirical calculation of the aerodynamic parameters, the 3D model of Extra300 is established and the structural size is measured. The characteristic parameters of the aircraft like mass *m*, position of center of gravity $\left(x_{G},y_{G},z_{G}\right)$ and moment of inertia *I* are tested successively on a Mass & Gravity Center Test Board and a Moment of Inertia Turntable. Firstly, by setting each aircraft’s axis along the central axis of the Test Board, shown in the left part of Figure 3, *m* and $\left(x_{G},y_{G},z_{G}\right)$ are obtained based on the moment balance principle. It is tested that the thrust from the propeller $F_{T}$ points nearly through the center of gravity. Then, the aircraft will be installed on the turntable. Make sure that the rotation axis of the turntable points right through the obtained gravity center, shown in the right part of Figure 3. Moment of inertia is calculated from the rotation period tested by an optoelectronic switch. The rotation is generated by the turntable and slowed down by a spring device. Before the whole test, a standard cylinder with known mass (about 3 kg) and moment of inertia (about 0.00058 kg · m${}^{2}$) is used to calibrate the parameters of the two test tables, like the elastic coefficient of the spring device. Together with the above, the empirical aerodynamic derivatives calculated according to Section 2 are listed in Table 2.

The airborne sensors and devices are shown in Figure 2. A XSENS MTi-G module is chosen, which owns a mature integrated navigation system of INS/GPS. It also contains an Attitude and Heading Reference System (AHRS) processor. XSENS MTi-G deploys MEMS inertial sensors in 9 axises, in which the outputs of gyroscopes own the $1^{{}^{\circ}}/s$ bias stability and $0.05^{{}^{\circ}}/s$ noise density, the accelerometers have the 0.02 m/s${}^{2}$ bias stability and 0.002 m/s${}^{2}$ noise density, the magnetic sensors has 0.1 m Gauss bias stability and 1.5 m Gauss noise density. MTi-G provides not only the outputs of different inertial sensors but also the integrated navigation results of attitude, velocity and position. The accuracy of the integrated position and velocity is less than 2.5 m and 0.1 m/s respectively. The dynamic accuracy of the pitch/roll angle is less than $1^{{}^{\circ}}$ and that of the heading angle is less than $2^{{}^{\circ}}$. Thus, the complex initial alignment process for INS or ADM is left out. The data output rate of XSENS is usually set to be 100 Hz [30].

The data of MTi-G is collected through a data acquisition board. As shown in the picture, the collecting board, placed in the cabin, has 2 layers, both of which has the core of DSP-BF506F. The lower board is in charge of collecting the data of all the sensors and transmitting to the upper board. The upper board will collect the data from the lower board and that of the signal receiver of 4 channels transmitted from the controller, then output all of them to a data recorder. The data recorder is recommended, rather than the radio transmission, to avoid the loss of data or electromagnetic interference. Furthermore, the upper board is responsible for the switching of the flight mode. In the tests, the aircraft is controlled remotely by a controller, namely the RC mode [31,32].

**Figure 4:**
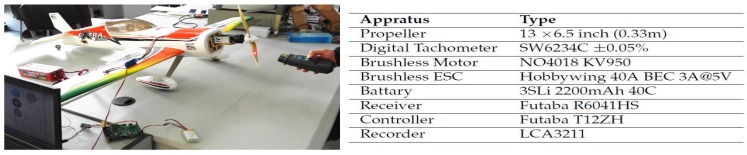
Rotation Rate Test.

Aiming at the verification of the accuracy of the aerodynamic parameters, the aircraft dynamic and kinematic model should be calculated with the input data of the actuators in the real flight. So the correspondence between the width of PWM signal , $d_{PWM}$, transmitted from the controller and the actuators’ inputs, namely the rotation rate *n* and the deflection angles δ, has to be derived. Arifianto [8] establishes the model by testing the force of the propeller corresponding to the rotation rate, but the airspeed is ignored in the static experiment. Herein, the ”n-$d_{PWM}$” relationship is constructed directly, and the thrust will be calculated with the equation in Table 1. An experiment of testing the rotation rate of the propeller is firstly carried out, and the test and the relevant experimental apparatus is shown in Figure 4. The PWM signal width of the channel of the throttle $d_{PWM}$ ranges from 2202 to 3879. The rotation rate of the propeller is measured by a handheld digital tachometer corresponding to the different position of the joystick, which is limited to 70% of the full range for safety. After several tests and second order polynomial fit, the equation could be obtained in (23), and the maximal rotation rate could reach 8966.3 RPM, namely 149.4 r/s. Then, the relationship between the deflection angle of the control surfaces (measured by a digital goniometer) and the PWM width is fitted linearly in (24)–(26).

(23) $$n_{P}=\left(-0.001571d_{n}^{2}+13.18d_{n}-21300\right)/60$$

(24) $$\delta_{e}{(}^{{}^{\circ}})=\left\{\begin{array}{c} 49.2480-0.0162d_{e}\;\;\;\;\;\;\;\;2202\leq d_{e}\leq 3029\\ 60.1920-0.0198d_{e}\;\;\;\;\;\;\;\;3029<d_{e}\leq 3879 \end{array}\right.$$

(25) $$\delta_{r}{(}^{{}^{\circ}})=\left\{\begin{array}{c} 60.7800-0.0200d_{r}\;\;\;\;\;\;2200\leq d_{r}\leq 3040\\ 60.7800-0.0200d_{r}\;\;\;\;\;\;3040<d_{r}\leq 3879 \end{array}\right.$$

(26) $$\delta_{a}{(}^{{}^{\circ}})=\left\{\begin{array}{c} 55.5840-0.0180d_{a}\;\;\;\;\;\;\;2199\leq d_{a}\leq 3085\\ 58.6720-0.0190d_{a}\;\;\;\;\;\;\;3085<d_{a}\leq 3878 \end{array}\right.$$

## 5. Flight Test and Analysis

**Figure 5:**
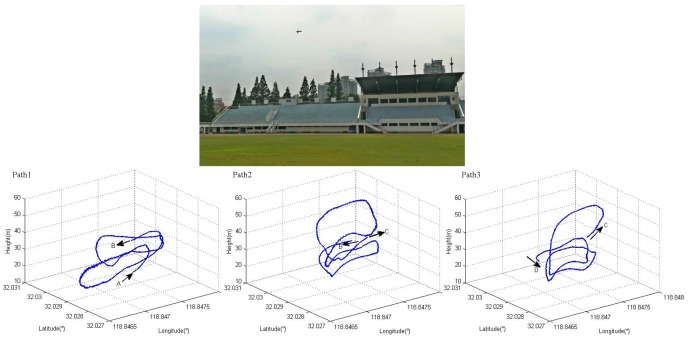
Flight Test.

Real flight tests were carried out in an open playground in Figure 5. The aircraft Extra300 was basically set to circle around above the field, controlled by a pilot on the ground. Several flight tests were completed and one of the experiment data was chosen to identify the aerodynamic derivatives, and then to verify the improvement compared with that calculated by the semi-empirical method. The 3D flight path is shown in Figure 5. It is truncated from the whole flight, lasting 3 min. To make it more clear to recognize the changing of the path, it is decomposed into three parts, from point A to D. In the flight route, there exists circling, slight climbing and diving, accelerating and decelerating and other maneuvering, which contribute to the observation. Besides, the measured speed is estimated as ground speed not the airspeed as the wind is neglected.

**Figure 6:**
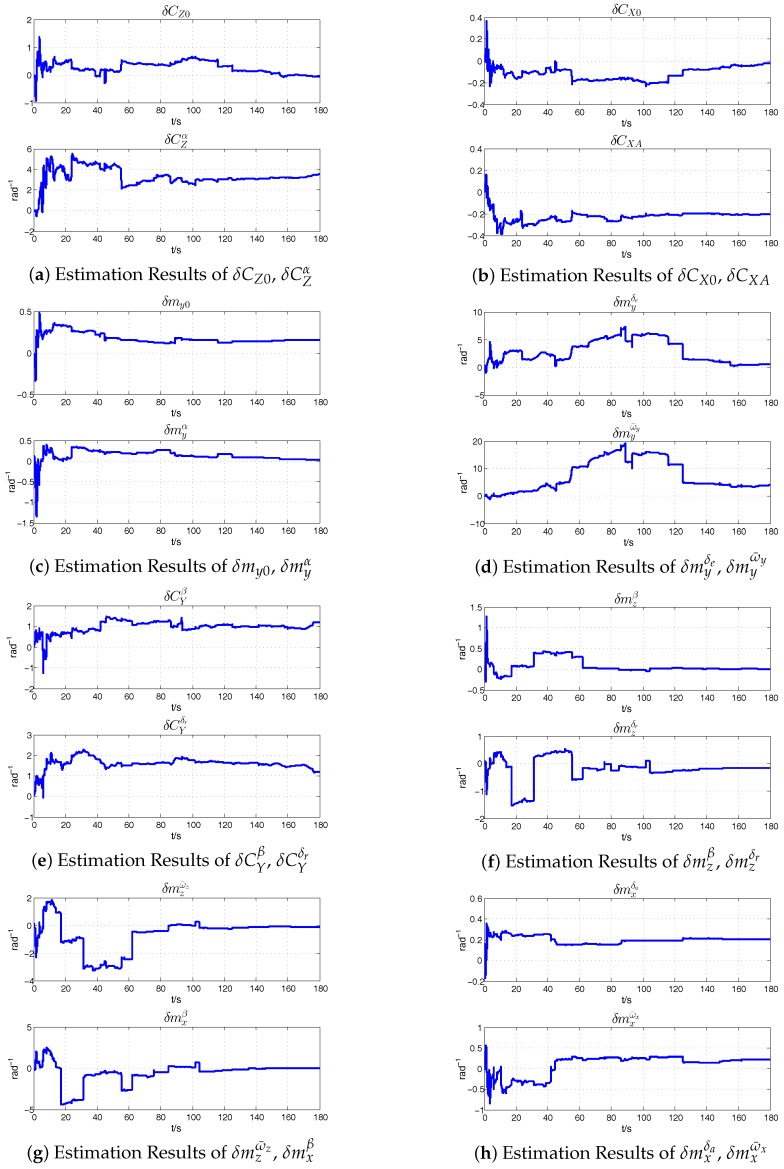
Aerodynamic Parameters Error Estimation Results.

The identification was operated according to the analysis in Section 3. The data fusion frequency is 10 Hz and the standard deviation of white noise is set according to the precision of the INS/GPS navigation results. The estimation results of the error of aerodynamic parameters are shown in Figure 6a–h, and all of them could converge to a certain range. Some coefficients, like $\delta m_{y}^{\delta_{e}}$, $\delta m_{y}^{{\bar{\omega }}_{y}}$, $\delta m_{z}^{\delta_{r}}$, $\delta m_{x}^{{\bar{\omega }}_{x}}$, converge piece-wisely. It may be caused by the changing of the real-time flight condition, and it matters slightly in this estimation process. The average of the estimation results in the converging section are calculated and approximated, shown in Table 3. Then, the estimated errors of the aerodynamic derivatives are subtracted or compensated from the initial derivatives calculated by semi-empirical method, listed in Table 2, thus further correcting the aircraft model.

**Figure 7:**
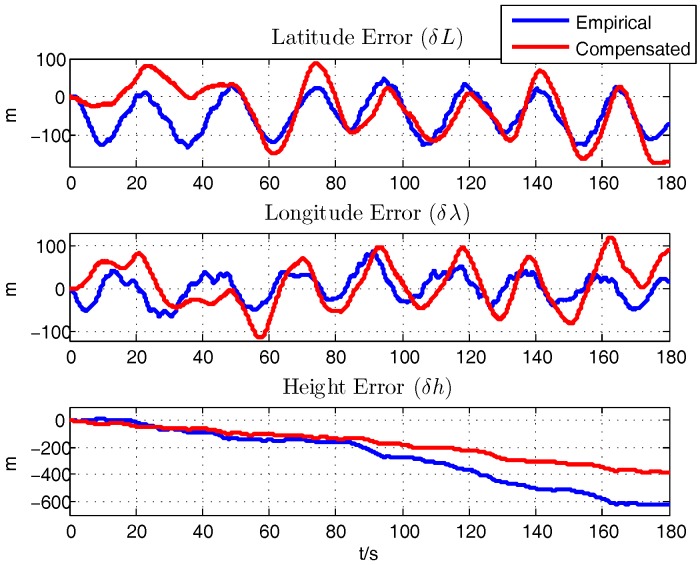
Position Error.

**Figure 8:**
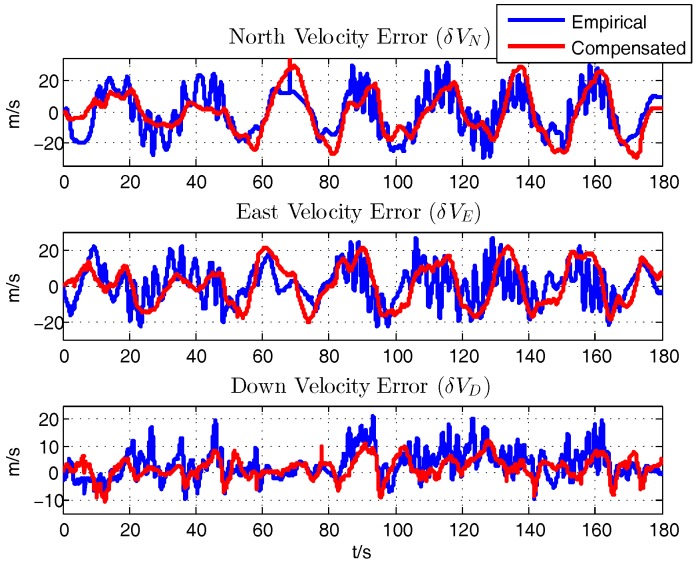
Velocity Error.

**Figure 9:**
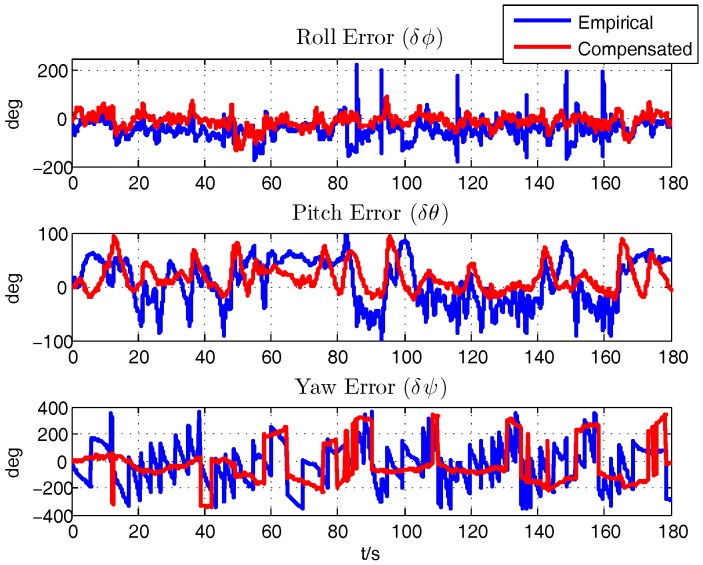
Attitude Error.

With the initial motion states provided by the INS/GPS integration in XSENS MTi-G, the navigation results from the integration of the aircraft motion equations of (14)–(16) is calculated. By comparing with the INS/GPS navigation results, on one hand, the rationality of the aerodynamic parameters computed by the semi-empirical method will be proved, on the other hand, the effect of latter compensation process is also verified, which is a unique method to validate the correctness of the calculation and identification results. Figure 7, Figure 8 and Figure 9 show the comparison of the errors of navigation results between the empirically calculated model and the compensated one by subtracting the INS/GPS navigation results.

Firstly, it is demonstrated that the semi-empirically calculated parameters based on aerodynamics are rational and useful, which is the premise for further analysis. After the compensation of the error of the aerodynamic derivatives, there shows an improvement in the navigation results. Specifically, the error of the latitude and longitude of the two processes are similar, and it is calculated that the average error of the latitude and longitude are about −39.1824 m and 5.5703 m respectively. The average of height error decreases to about 177.6206 m from 272.3769 m after the identification. The average errors of the horizontal velocity are alike, namely $\delta V_{n}$, $\delta V_{e}$ are about −0.9853 m/s, 0.8024 m/s respectively while $\delta V_{d}$ changes from 3.1785 m/s to 1.8770 m/s. Although there exists just a slight improvement in the horizontal velocity, it is obviously figured that the compensated model are more stable and owns a better tendency compared with the INS/GPS results. The average error of roll angle decreases from −44.4609${}^{{}^{\circ}}$ to−11.1175${}^{{}^{\circ}}$. The average error of pitch angle, around 12.6113${}^{{}^{\circ}}$, are similar but more stable. Yaw angle, with the average error of −26.4751${}^{{}^{\circ}}$, are more rational and precise after the compensation as the wide swing are taken out.

**Figure 10:**
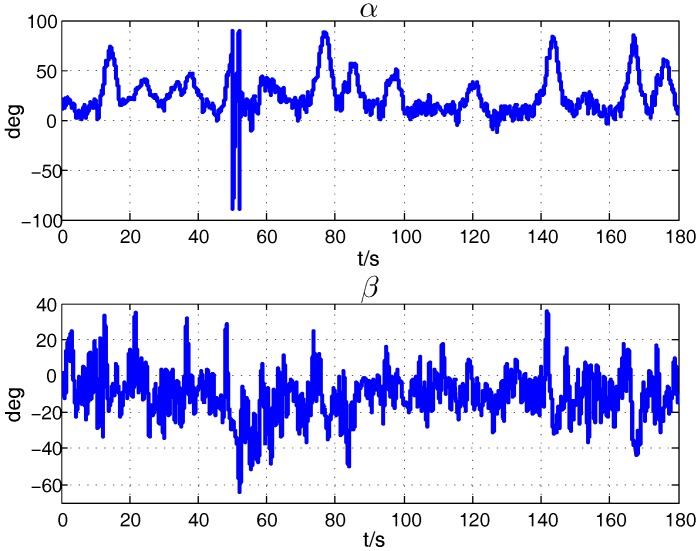
Aerodynamic Angle in the Flight.

Despite the improvement after the correction process, differences still exist between the compensated aircraft model and the precise integration results. The model of the rotation rate of propeller or the deflection angle of the actuators maybe inaccurate, but it has little effect on the navigation results according to the experiment. It is largely caused by the inaccuracy of the identification of aerodynamic parameters. The flight condition, like the large variation of the aerodynamic angle, will influence the estimation precision. It could be seen from the changing of α, β in Figure 10. As seen from the pictures, it doesn’t match the small angle-of-attack assumption sometimes, so some aerodynamic derivatives are no longer constants. Compared with the navigation error in Figure 8, the error just becomes larger when the angle-of-attack increases largely. The phenomenon also take place in other flight tests, and the estimation results of the parameters’ error are a little different in different flight sorties concerning the different flight condition. To address the problem, the nonlinear aerodynamic derivatives should be taken into account as the simplified modeling of the coefficients and derivatives leads to the uncertainty. For example, the slope of lift curve $C_{Z}^{\alpha }$ is variant, and some nonlinear derivatives associated with α, β should be augmented to the states to be estimated. The variant coefficients should be estimated precisely and compensated piece-wisely in a real time. Furthermore, more sensors measuring the aerodynamic angles α, β and the airspeed should be added to take the changing circumstances into account.

## 6. Conclusions

The paper mainly studied the method of calculating the aerodynamic parameters of the small-scaled fixed-wing aircraft, and it is an important part in the establishment of the aircraft dynamic and kinematic model. It contains 2 sequential processes:

(1) Prepared with the related structural parameters and the position of gravity center, the aerodynamic derivatives are semi-empirically calculated based on fundamental aerodynamics. The simplified structure and the specific flight condition reduce the complexity. So, the aerodynamic analysis could be operated in a fast, effective and low-cost way to originally provide a serial rational parameters. The paper introduces and analyzes the entire course of the calculating of the longitudinal and lateral derivatives, and it is helpful for the modeling of all the other similar small aircraft;
(2) The initially obtained parameters is further identified or compensated with the real flight data. Given the airborne MEMS-based inertial sensors with finite precision, the relatively accurate velocity and attitude from the integration of INS/GPS was employed as observations to estimate the error of the aerodynamic parameters. It may have the problem of lack of observations, but it could be solved with the abundant maneuvering flight. Then, the aerodynamic parameters could be corrected with the estimated error of the derivatives and the aircraft model could be more accurate and reliable.

The real flight test demonstrates a promising result of the above 2 methods. Abide by the traditional aerodynamics and the empiricism from flight database, a rational aircraft model is constructed. Although about 20% or more error exists in the aerodynamic coefficients, it still provides a basic prototype or a baseline model. During the circling flight test, the controller adjusted the model aircraft all the time, thus providing the required maneuvers to increase the observability of all the derivatives in the identification process. And the accuracy of the compensated parameters was verified by integrating the aircraft dynamic and kinematic model, then comparing with the INS/GPS integration results, which is different from the traditional verification. The accuracy of the method synthesizes the precision of the model itself including the structural and aerodynamic part as well as the precision of the actuators’ inputs. But the correctness of the aerodynamic parameters affect predominantly.

As discovered from the tests, the ideal aerodynamic parameters, namely the true values, are hard to reach. Firstly, the inaccuracy of the structural parameters, the possible shifting of the position of the center of gravity, and the related assumptions and simplifications could make an effect on the semi-empirical analysis. Then, the variant flight condition makes it more difficult for the filter to identify the variant parameters correctly and instantaneously, especially encountering the disturbance of the wind or gust. In the subsequent study or experiment, more aerodynamic sensors, like the low-cost or homemade vanes measuring the angle-of-attack and the side-slip angle, will be equipped to take the wind into consideration. Then, the relatively accurate aircraft model are expected to be used as an assistance for the fast divergent pure INS in the future research. 

## Figures and Tables

**Table 1:** Formulas of the Forces & Moments.

Forces/Moments	Formulas
Thrust $F_{T}$	$F_{T}=\rho n_{P}^{2}D^{4}\left[C_{FT1}+C_{FT2}\frac{V_{a}Ma}{D\pi n_{P}}+C_{FT3}{\left(\frac{V_{a}Ma}{D\pi n_{P}}\right)}^{2}\right]$
Lift *Z*	$Z=QSC_{Z}\;\;\;\;\;\;C_{Z}=C_{Z0}+C_{Z}^{\alpha }\alpha +C_{Z}^{\delta_{e}}\delta_{e}$
Drag *X*	$X=QSC_{X}\;\;\;\;\;C_{X}=C_{X0}+C_{Xi}=C_{X0}+AC_{Z}^{2}$
Pitch Moment $M_{y}$	$M_{y}=QS\bar{c}m_{y}\;\;\;m_{y}=m_{y0}+m_{y}^{\alpha }\alpha +m_{y}^{\delta_{e}}\delta_{e}+m_{y}^{{\bar{\omega }}_{y}}\frac{\omega_{y}\bar{c}}{V_{a}}$
Side Force *Y*	$Y=QSC_{Y}\;\;\;\;\;\;C_{Y}=C_{Y}^{\beta }\beta +C_{Y}^{\delta_{r}}\delta_{r}$
Yaw Moment $M_{z}$	$M_{z}=QSlm_{z}\;\;\;m_{z}=m_{z}^{\beta }\beta +m_{z}^{\delta_{r}}\delta_{r}+m_{z}^{{\bar{\omega }}_{z}}\frac{\omega_{z}l}{2V_{a}}+m_{z}^{{\bar{\omega }}_{x}}\frac{\omega_{x}l}{2V_{a}}$
Roll Moment $M_{x}$	$M_{x}=QSlm_{x}\;\;\;m_{x}=m_{x}^{\beta }\beta +m_{x}^{\delta_{r}}\delta_{r}+m_{x}^{\delta_{a}}\delta_{a}+m_{x}^{{\bar{\omega }}_{z}}\frac{\omega_{z}l}{2V_{a}}+m_{x}^{{\bar{\omega }}_{x}}\frac{\omega_{x}l}{2V_{a}}$

**Table 2:** Extra300 Parameters.

Structural Parameters	S=0.285 m${}^{2}$, l=1.2 m, $\bar{c}=0.24$ m		
Propeller Parameters	ρ=1.22 kg/m${}^{3}$, D=0.33 m $C_{FT1}=0.0842,C_{FT2}=-0.136,C_{FT3}=-0.928$		
Inertia Parameters	m=1.72 kg, $x_{G}=0.323$ m, $y_{G}\approx 0$ m, $z_{G}\approx 0$ m $I=\left[\begin{array}{ccc} 0.001393 & 0 & 0\\ 0 & 0.003543 & 0\\ 0 & 0 & 0.003631 \end{array}\right]$ kg·m${}^{2}$		
Aerodynamic Parameters	$C_{Z}$	$C_{X}$	$m_{y}$
	$C_{Z0}=0.1601$	$C_{X0}=0.01602$	$m_{y0}=0.05267$
	$C_{Z}^{\alpha }=4.4851$ rad${}^{-1}$	$C_{XA}=0.1347$	$m_{y}^{\alpha }=-0.1195$ rad${}^{-1}$
	$C_{Z}^{\delta_{e}}=0.5901$ rad${}^{-1}$		$m_{y}^{\delta_{e}}=-2.8648$ rad${}^{-1}$
			$m_{y}^{{\bar{\omega }}_{y}}=-6.4099$ rad${}^{-1}$
	$C_{Y}$	$m_{z}$	$m_{x}$
	$C_{Y}^{\beta }=-0.7076$ rad${}^{-1}$	$m_{z}^{\beta }=-0.007517$ rad${}^{-1}$	$m_{x}^{\beta }=-0.05094$ rad${}^{-1}$
	$C_{Y}^{\delta_{r}}=-0.3893$ rad${}^{-1}$	$m_{z}^{\delta_{r}}=-0.2152$ rad${}^{-1}$	$m_{x}^{\delta_{a}}=-0.3022$ rad${}^{-1}$
		$m_{z}^{{\bar{\omega }}_{z}}=-0.5053$ rad${}^{-1}$	$m_{x}^{\delta_{r}}=-0.06492$ rad${}^{-1}$
		$m_{z}^{{\bar{\omega }}_{x}}=-0.05272$ rad${}^{-1}$	$m_{x}^{{\bar{\omega }}_{x}}=-0.3968$ rad${}^{-1}$
			$m_{x}^{{\bar{\omega }}_{z}}=-0.007$ rad${}^{-1}$

**Table 3:** Parameter Error Estimation Results.

Parameter	Result	Parameter	Result
$\delta C_{Z0}$	0.000	$\delta C_{Y}^{\beta }$	1.000
$\delta C_{Z}^{\alpha }$	3.000	$\delta C_{Y}^{\delta_{r}}$	1.600
$\delta C_{Z}^{\delta_{e}}$	0.000	$\delta m_{z}^{\beta }$	0.000
$\delta C_{X0}$	−0.100	$\delta m_{z}^{\delta_{r}}$	−0.166
$\delta C_{XA}$	−0.200	$\delta m_{z}^{{\bar{\omega }}_{z}}$	−0.110
$\delta m_{y0}$	0.150	$\delta m_{z}^{{\bar{\omega }}_{x}}$	0.147
$\delta m_{y}^{\alpha }$	0.100	$\delta m_{x}^{\beta }$	0.000
$\delta m_{y}^{\delta_{e}}$	1.000	$\delta m_{x}^{\delta_{a}}$	0.200
$\delta m_{y}^{{\bar{\omega }}_{y}}$	5.000	$\delta m_{x}^{\delta_{r}}$	0.000
		$\delta m_{x}^{{\bar{\omega }}_{x}}$	0.200
		$\delta m_{x}^{{\bar{\omega }}_{z}}$	0.593

## Nomenclature

***Abbreviation***	
ADM	Aircraft Dynamic Model
AMM	Aircraft Motion Model
CFD	Computational Fluid Dynamics
EKF	Extended Kalman Filter
GPS	Global Positioning System
IMU	Inertial Measurement Unit
INS	Inertial Navigation System
MEMS	Micro-Electro Mechanical Systems
MLE	Maximum Likelihood Estimation
PMW	Pulse Width Modulation
UAV	Unmanned Aerial Vehicle
UKF	Unscented Kalman Filter
***Coordinate***	
g	Earth-surface inertial reference frame pointing North, East, Down
b	Aircraft-body coordinate frame
a	Wind coordinate frame
$C_{g}^{b}$	Coordinate rotation matrix from *g* to *b*
$C_{a}^{b}$	Coordinate rotation matrix from *a* to *b*
***Aircraft***	
*m*	Total mass, kg
*I*	Moment of inertia, kg·m${}^{2}$
$x_{G},y_{G},z_{G}$	Position of center of gravity, m
$x_{F}$	Aerodynamic focus, m
$x_{pW},x_{pB},x_{pWB}$	Aerodynamic center of wing, body and wing-body, m
$x_{pHT},x_{pHT\delta_{e}}$	Aerodynamic center of horizontal tail and elevator, m
*l*	Wing span, m
$\bar{c}$	Mean aerodynamic chord, m
*S*	Reference area, m${}^{2}$
$S_{CW}$	Cantilever wing area, m${}^{2}$
$S_{B}$	Fuselage maximum cross-sectional area, m${}^{2}$
$S_{HT}$	Horizontal tail area, m${}^{2}$
λ	Aspect ratio
$\bar{D}$	Fuselage diameter-to-wing span ratio
*D*	Diameter of propeller, m
$\varphi_{W},\varphi_{S}$	Incidence angle of wing, horizontal tail, degree
$K_{\alpha \alpha },K_{\phi 0}$	Wing-body interference factor
${\left(K_{\alpha \alpha }\right)}_{HT},{\left(K_{\phi 0}\right)}_{HT}$	Horizontal tail-body interference factor
$F_{T}$	Thrust of propeller, N
$F_{A}={\left[X,Y,Z\right]}^{T}$	Aerodynamic forces in wind coordinates, Drag, Side Force and Lift, N
$M_{b}={\left[M_{x},M_{y},M_{z}\right]}^{T}$	Aerodynamic moments in body coordinates, Roll, Pitch and Yaw moment, N· m
$C_{FT1},C_{FT2},C_{FT3}$	Dimensionless thrust coefficients
$C_{X},C_{Y},C_{Z}$	Dimensionless aerodynamic-force coefficients
$m_{x},m_{y},m_{z}$	Dimensionless aerodynamic-moment coefficients
$V_{a}$	Airspeed, m/s
α	Angle-of-attack, rad
β	Angle of sideslip, rad
ε	Angle of downwash, rad
ρ	Air density, kg/m${}^{3}$
*Q*	Dynamic pressure, Pa
$k_{HT}$	Airflow block factor
$\delta_{e},\delta_{r},\delta_{a}$	Deflection angle of elevator, rudder and aileron, rad
$n_{P}$	Revolutions per second of propeller, r/s
$d_{n},d_{e},d_{r},d_{a}$	PWM width corresponding to the channel of thrust, elevator, rudder and aileron
ϕ,θ,ψ	Roll, pitch, yaw angle rad
$V^{b}={\left[u,v,w\right]}^{T}$	Body axis velocities, m/s
$\omega^{b}={\left[p,q,r\right]}^{T}$	Body axis angular rates, rad/s
